# Core genome components and lineage specific expansions in malaria parasites *Plasmodium*

**DOI:** 10.1186/1471-2164-11-S3-S13

**Published:** 2010-12-01

**Authors:** Hong Cai, Jianying Gu, Yufeng Wang

**Affiliations:** 1 Department of Biology, University of Texas at San Antonio, San Antonio, TX 78249, USA; 2 Department of Biology, College of Staten Island, City University of New York, Staten Island, NY 10314, USA; 3 South Texas Center for Emerging Infectious Diseases, University of Texas at San Antonio, San Antonio, TX 78249, USA

## Abstract

**Background:**

The increasing resistance of *Plasmodium,* the malaria parasites, to multiple commonly used drugs has underscored the urgent need to develop effective antimalarial drugs and vaccines. The new direction of genomics-driven target discovery has become possible with the completion of parasite genome sequencing, which can lead us to a better understanding of how the parasites develop the genetic variability that is associated with their response to environmental challenges and other adaptive phenotypes.

**Results:**

We present the results of a comprehensive analysis of the genomes of six *Plasmodium* species, including two species that infect humans, one that infects monkeys, and three that infect rodents. The core genome shared by all six species is composed of 3,351 genes, which make up about 22%-65% of the genome repertoire. These components play important roles in fundamental functions as well as in parasite-specific activities. We further investigated the distribution and features of genes that have been expanded in specific Plasmodium lineage(s). Abundant duplicate genes are present in the six species, with 5%-9% of the whole genomes composed lineage specific radiations. The majority of these gene families are hypothetical proteins with unknown functions; a few may have predicted roles such as antigenic variation.

**Conclusions:**

The core genome components in the malaria parasites have functions ranging from fundamental biological processes to roles in the complex networks that sustain the parasite-specific lifestyles appropriate to different hosts. They represent the minimum requirement to maintain a successful life cycle that spans vertebrate hosts and mosquito vectors. Lineage specific expansions (LSEs) have given rise to abundant gene families in *Plasmodium.* Although the functions of most families remain unknown, these LSEs could reveal components in parasite networks that, by their enhanced genetic variability, can contribute to pathogenesis, virulence, responses to environmental challenges, or interesting phenotypes.

## Background

Malaria affects approximately 300 million people worldwide and kills between 1 and 1.5 million people every year. It has been largely controlled by effective medicines until recently, but malaria parasites have gradually developed resistance to multiple drugs and pose an increasingly important health threat.

The causative agents of malaria are protozoan parasites in the genus *Plasmodium.* Four species of *Plasmodium* cause malaria in humans: *Plasmodium falciparum, P.vivax, P. ovale,* and *P. malaria. P. falciparum* is the most widespread and devastating one; if untreated it can be fatal. Other species from this genus are known to infect rodents and non-human primates.

The complete sequencing of various malaria parasite genomes has brought new hope for the discovery of new antimalarial targets [1-5]. Before the genome of *P.falciparum* was sequenced, only about 20 proteins had been characterized. Genome sequencing revealed over 5,400 open reading frames (ORFs) in *P. falciparum.* Successful application of the genomic analysis approach has already lead to the discovery of potential vaccine targets such as *P. falciparum* erythrocyte membrane protein families (PfEMPs) [6,7] and drug targets such as a 1-deoxy-D-xylulose 5-phosphate (DOXP) reductoisomerase [8] and a catalog of proteases that may play important roles in parasite development and invasion [9-11]. Comparative genomics has also shed significant light on the mechanisms of drug resistance involving transporter proteins [12]. The release of the genome data has also made it possible to carry out large scale expression analysis at the transcriptome and proteome levels. Microarray and proteomic experiments have revealed interesting expression patterns of gene products under specific temporal and spatial conditions [13-19], providing a blueprint for a systems level study of gene regulatory networks, protein-protein networks and metabolic networks [20-22], and representing the beginning of a new era of systems biology in malaria research.

Within the scheme of systems biology, one of the interesting questions is how parasites develop genetic variability that can be tied to their response to environmental challenges and other adaptive phenotypes.

In this study, we propose to explore the genome context and systems evolution of six model species of *Plasmodium: P.falciparum and P. vivax* are the model system for human parasites, and cause the first and second most severe forms of human malaria; *P. knowlesi* used to be considered as a model system for the simian parasite whose natural mammalian host is the Macaque monkey, however, increasing evidence shows that naturally occurring *P. knowlesi-induced* human malaria is not rare [5,23]; *P. yoelii yoelii, P. berghei,* and *P. chabaudi* are the model systems of rodent parasites which have been used widely and successfully to complement research on human malaria parasites.

We focus on two fundamental questions: (1) What are the common components in these six malaria parasites? As they all have evolved a successful parasite lifestyle, the core genome structure may reveal critical adaptive features. (2) What are the lineage specific components in each species? In particular, we are interested in genes or gene families that have been largely expanded in one or several unique lineages. We show that the core genome and lineage-specific expanded genome components involve genes that are tied to pathogenesis and virulence mechanisms as well as in the fundamental life cycle of *Plasmodium* species.

## Results and discussion

### The core genome of six *Plasmodium* species

#### (1) The core genome is comprised of 3,351 othologous genes

The orthoMCL analysis revealed that the core genome of the six *Plasmodium* species we examined is comprised of 3,351 othologous genes (Table 1). The catalog of the core genome is summarized in Additional file 1. The proportions of core genome components in the two human malaria parasites *(P. falciparum* and *P. vivax)* were very similar (approximately 61%). The simian parasite *P. knowlesi* has a slightly larger genome and a higher proportion of the core genome (66%). The three rodent species seem to have more diverse genomes; only about 22-42% of the genes encode core components. The numbers of the predicted ORFs in the *P. berghei* genome (12,235) and in the *P. chabaudi* genome (15,007) are relatively larger than those in the other four species due to the fragmented nature of the sequence data and incomplete annotation of these genomes [17], therefore results for these species must be seen as preliminary.

**Table 1:** The core genome components and lineage specific genes in six *Plasmodium* species. The inter-genomic search yielded a core genome comprised of 3,351 orthologous proteins

Strains	No. Genes in genome	% core in genome	No. Families with LSE		No. LSE genes	% LSEs in genome

			Lineage-unique	Typical LSE		
*P. berghei*	12,235	27.39	111	323	960	7.84
*P. chabaudi*	15,007	22.33	176	379	1342	8.94
*P. falciparum*	5,460	61.37	36	13	510	9.34
*P. knowlesi*	5,110	65.57	12	14	293	5.73
*P. vivax*	5,432	61.69	45	21	488	8.98
*P. yoelii yoelii*	7,861	42.63	62	65	553	7.03

Interestingly, 1,079 (33%) of the 3,351 orthologous clusters in the core genome were predicted to fall into at least one Gene Ontology class, while the remaining 2,272 (67%) appear to have no identifiable ontology functions. This is consistent with the fact that at least 60% of the 5,460 ORFs in the best-annotated *Plasmodium* species, *P. falciparum,* were annotated as "hypothetical protein", indicating that no reliable functional prediction/characterization was available [4].

#### (2) Core genome components involved in fundamental biological processes in *Plasmodium*

Despite their different host specificities, the six *Plasmodium* species preserve the common components that are essential for their fundamental biology (see examples in Table 2).

**Table 2:** The core genome components in six Plasmodium species involved in fundamental cellular processes.

Function description	Examples of GO classes	Orthologous families
**Replication**	GO:0003688 (DNA replication origin binding)	ORTHOMCL1162
		ORTHOMCL2123

	GO:0003887 (DNA-directed DNA polymerase activity)	ORTHOMCL2751
		ORTHOMCL61
		ORTHOMCL2153
		ORTHOMCL593
		ORTHOMCL2507

	GO:0005663 (DNA replication factor C complex)	ORTHOMCL1738
		ORTHOMCL1861
		ORTHOMCL443
		ORTHOMCL513
		ORTHOMCL1911
		ORTHOMCL2437
		ORTHOMCL3328

	GO:0005662 (DNA replication factor A complex)	ORTHOMCL683

**Transcription**	GO:0000122 (negative regulation of transcription from RNA polymerase II promoter)	ORTHOMCL190

	GO:0000126 (transcription factor TFIIIB complex)	ORTHOMCL2349
		ORTHOMCL802

	GO :0016251 (general RNA polymerase II transcription factor activity)	ORTHOMCL2179

	GO:0003702 (RNA polymerase II transcription factor activity)	ORTHOMCL1522
		ORTHOMCL3420

	GO:0003712 (transcription cofactor activity)	ORTHOMCL3015

	GO:0003700 (transcription factor activity)	ORTHOMCL1875
		ORTHOMCL3398
		ORTHOMCL880
		ORTHOMCL2851
		ORTHOMCL2947

**Translation**	GO:0006412 (translation)	ORTHOMCL1544
		ORTHOMCL3343
		ORTHOMCL1471
		ORTHOMCL1516
		ORTHOMCL1698
		ORTHOMCL2550
		ORTHOMCL1856

	GO:0003743 (translation initiation factor activity)	ORTHOMCL1832
		ORTHOMCL1842
		ORTHOMCL2705
		ORTHOMCL3178
		ORTHOMCL2122
		ORTHOMCL940
		ORTHOMCL3423

	GO:0003746 (translation elongation factor activity)	ORTHOMCL350
		ORTHOMCL1193
		ORTHOMCL2152
		ORTHOMCL2232
		ORTHOMCL1803
		ORTHOMCL516
		ORTHOMCL1744

	GO:0006449 (regulation of translational termination)	ORTHOMCL2253

**Repair**	GO:0006289 (nucleotide-excision repair)	ORTHOMCL444

	GO:0000724 (double-strand break repair via homologous recombination)	ORTHOMCL1863

	GO:0006302 (double-strand break repair)	ORTHOMCL867

	GO:0006281 (DNA repair)	ORTHOMCL543
		ORTHOMCL1058

**Cell motion**	GO:0007017 (microtubule-based process)	ORTHOMCL2981
		ORTHOMCL1078

	GO:0003777 (microtubule motor activity)	ORTHOMCL2241
		ORTHOMCL2737

	GO:0007018 (microtubule-based movement)	ORTHOMCL1635
		ORTHOMCL2737
		ORTHOMCL1944

	GO:0030048 (actin filament-based movement)	ORTHOMCL278
		ORTHOMCL1146
		ORTHOMCL428

Abundant orthologous families are involved in genetic information processing: replication, transcription and translation. None of these processes in malaria parasites are fully understood. For example, it is believed that the transcriptional regulation of malaria parasites is very complex, as it must adapt to different developmental processes in their vertebrate hosts and invertebrate mosquito vectors such as sexual development, parasite invasion, and antigenic variation. However, to date, only a small number of general transcription factors have been identified [24]. Recently, microarray expression and machine learning approaches have revealed putative cis-regulatory promoters that may be associated with specific transcription factors [25,26]. The core genome of these six *Plasmodium* species suggests that the basic transcriptional machinery includes the essential enzymes, general transcription factors, and positive and negative transcriptional cofactors. Similarly, the common elements of the translational machinery are also present in the core genome, including orthologous clusters that regulate the initiation, elongation, and termination of the processes. Associated with translation, we also observe that the RNA splicesome is conserved in the six *Plasmodium* genomes: orthologous clusters are predicted to belong to the GO classes of small nucleolar ribonucleoprotein complex (GO:0005732), spliceosome assembly (GO:0000245), RNA splicing factor activity, transesterification mechanism (GO:0031202), spliceosome (GO:0005681), snRNP U1 (GO:0005685), and snRNP U2 (GO:0005686). The core genome also includes components that are essential for repair mechanisms and cell motion.

#### (3) Core genome components involved in cellular processes related to the parasite lifestyle

In addition to the genes or gene products that are required for fundamental biology, we are particularly interested in the core genome components that are pertinent to parasite-specific lifestyles. Representative functional classes of orthologous clusters are shown in Table 3.

**Table 3:** Representative cellular processes related to parasite specific lifestyle that are commonly present in six *Plasmodium* genomes.

Function description	Examples of GO classes	Orthologous families
**Cell Cycle**	GO:0000079 (Regulation of cyclin-dependent protein kinase activity)	ORTHOMCL1356
		ORTHOMCL2659

	GO:0051726 (regulation of cell cycle)	ORTHOMCL703
		ORTHOMCL2129
		ORTHOMCL2139
		ORTHOMCL3332
		ORTHOMCL93
		ORTHOMCL1497
		ORTHOMCL2030
		ORTHOMCL3034

	GO:0045836 (positive regulation of meiosis)	ORTHOMCL1572

	GO:0007049 (cell cycle)	ORTHOMCL3532
		ORTHOMCL1160

	GO:0000082 (G1/S transition of mitotic cell cycle)	ORTHOMCL3162

**Signal transduction**	GO:0007266 (Rho protein signal transduction)	ORTHOMCL2354

	GO:0007165 (signal transduction)	ORTHOMCL3462
		ORTHOMCL3526
		ORTHOMCL3426
		ORTHOMCL1645
		ORTHOMCL710

	GO:0007186 (G-protein coupled receptor protein signaling pathway)	ORTHOMCL3024

	GO:0008426 (protein kinase C inhibitor activity)	ORTHOMCL2343

**Response to environmental challenges**	GO:0006979 (response to oxidative stress)	ORTHOMCL2680
		ORTHOMCL602
		ORTHOMCL2542
		ORTHOMCL1549
		ORTHOMCL2530
		ORTHOMCL1476
		ORTHOMCL3291
		ORTHOMCL3446
		ORTHOMCL2315

	GO:0006950 (response to stress)	ORTHOMCL3208

	GO:0009408 (response to heat)	ORTHOMCL2452
		ORTHOMCL2633
		ORTHOMCL112
		ORTHOMCL237
		ORTHOMCL702
		ORTHOMCL803
		ORTHOMCL1088
		ORTHOMCL1813
		ORTHOMCL3347
		ORTHOMCL1486
		ORTHOMCL2019
		ORTHOMCL3266
		ORTHOMCL700

**Pathogenesis**	GO:0009405 (pathogenesis)	ORTHOMCL2797

	GO:0030260 (entry into host cell)	ORTHOMCL106
		ORTHOMCL15
		ORTHOMCL2196
		ORTHOMCL2303

	GO:0042493 (response to drug)	ORTHOMCL3437
		ORTHOMCL780

	GO:0020035 (cytoadherence to microvasculature, mediated by parasite protein)	ORTHOMCL361
		ORTHOMCL41

One of the most important cellular processes that are critical for a successful life cycle in malaria parasites is cell cycle regulation. During the red blood cell stage, malaria parasites undergo atypical cell cycles. The entire genetic regulatory network of the cell cycle remains largely unknown [27-29]. Previously, we proposed a cell cycle network composed of 38 components using a Variational Bayesian expectation maximization (VBEM) approach based on comparative genomic prediction and microarray time-series expression profile [30]. This study confirmed that fifteen of the orthologous clusters in the *Plasmodium* core genome are members of the cell cycle network. For example, ORTHOMCL1356 and ORTHOMCL2659 may both be involved in cyclin-dependent kinase regulation (Table 3). The next step will be to place these orthologous genes in a network context.

In addition to the cell cycle, signal transduction also plays a role in other cellular networks. For example, at least one orthologous cluster (ORTHOMCL3024) is found in all six *Plasmodium* species and may participate in a G-protein coupled receptor (GPCR) protein signaling pathway. GPCRs have been attractive therapeutic targets for human diseases due to their versatile and critical roles in many signal transduction pathways. However, to date, no GPCR homolog has been identified in a *Plasmodium* genome, although Rab GTPases are found in the *P. falciparum* genome [4]. The core component ORTHOMCL3024 encodes a receptor for an activated C kinase homolog, named pfRACK, in *P. falciparum.* It has a single homolog in the other five *Plasmodium* species, all of which contain guanine nucleotide-binding motifs. It has been shown that pfRACK mRNA is expressed throughout the 48-hour red blood cell (RBC) cycle [13,19], and its protein product has been found in red blood cell membrane, and in the merozoite and trophozoite stages of the RBC cycle in several independent proteomics experiments [15,16]. Notably, it was previously reported that signaling via human erythrocytic GPCR regulated the entry of malaria parasites and a GPCR inhibitor blocked malaria infection [31], which makes GPCR agonists potential antimalarial targets. The existence of parasite proteins that may be involved in GPCR-like activities suggests that other parasite signaling proteins may be associated with host proteins to contribute to the parasite entry process.

Parasites, during their complex life cycles, also need to meet the challenges from various environmental signals. At least 23 orthologous clusters that play a role in the parasite responses to heat and stress, such as oxidative stress, are commonly shared in the six *Plasmodium* species.

Moreover, the core genome contains orthologous clusters that may be relevant to pathogenesis or virulence. Four othologous clusters (ORTHOMCL106, ORTHOMCL15, ORTHOMCL2196, ORTHOMCL2303) may be related to the host cell entry process. For example, ORTHOMCL106 includes 3 copies of Merozoite Surface Protein 7 (MSP7) precursor homologs (accession numbers MAL13P1.173, MAL13P1.174, and PF13_0197), and one hypothetical protein (PF13_0191) in *P. falciparum.* These four genes are tandemly located at adjacent positions in the same direction on Chromosome 13. They all seem to code for antigenic epitopes, and the three MSP7-like proteins were all expressed at the RBC surface [15]. MSP7 was reported to be expressed at the merozoite surface and associated with the MSP1 complex shed during RBC invasion [32]. Various copies of MSP7 homologs are present in other species (1 in *P. berghei, P. knowlesi, P. yoelii yoelii, 2* in *P. chabaudi,* 3 in *P. vivax),* suggesting that RBC entry requires similar surface proteins in all species.

ORTHOMCL2797 is another orthologous cluster that is predicted to be related to pathogenesis. It encodes a transmission-blocking target antigen s230 precursor (Pfs230) in *P. falciparum* and one single copy is present in the other five *Plasmodium* species. Pfs230 is expressed on the plasma membrane of parasite gametocytes in the human host and, after the parasites are taken up in a blood meal by a mosquito vector, it remains on the surface of the emerged gamete [33]. Transmission activity was found to be blocked when anti-Pfs230 antibodies were used, suggesting Pfs230 can be a potential vaccine target.

Two orthologous clusters might be related to the parasite's response to drugs. ORTHOMCL3437 contains one copy of chloroquine resistance transporter in each *Plasmodium* species; ORTHOMCL780 contains one copy of a multidrug resistance protein in five *Plasmodium* species, and 2 copies in *P. chabaudi.*

### Lineage specific expansions (LSEs) in *Plasmodium* species

The comparative genomic analysis of six *Plasmodium* species revealed genes that are specifically expanded in certain lineage(s). The emergence of multiple gene copies by duplication or lateral gene transfer in a specific lineage is known as a lineage specific expansion (LSE) event. Gene duplication has long been considered as a driving force for functional novelty as the duplicate copy can serve as a shield for the other copy with otherwise deleterious mutations to evolve novel functions under relaxed evolutionary constraints [34]. Parasites can also acquire new genes from other organisms via lateral gene transfer. The subsequent expansion of these new genes can increase the number of gene copies. LSEs are believed to be of critical importance to the evolution of genome plasticity as they provided opportunities for functional redundancy which could lead to the emergence of new functions [35].

A large number of duplicate genes have been identified in *Plasmodium.* Among them, abundant genes exhibit lineage specific expansions, accounting for approximately 5%-9% of the whole genomes (see Table 1 for the summary, and also see Additional file 2 for the detailed gene lists), suggesting that these parasite genomes have undergone frequent gene duplications that may confer advantages in selection. Two human malaria parasites, *P.falciparum* and *P*. *vivax* possess the largest proportion of LSEs. Three rodent parasite species *P. berghei, P. chabaudi* and *P. yoelii yoelii* have slightly smaller proportion of LSE genes than the human parasites, ranging from 7.03%-8.94%. *P. knowlesi,* however, contains significantly smaller number of duplicate genes, compared to the other five sibling species.

We observed two distinct patterns of LSE gene families in the *Plasmodium* genomes: (1) lineage-unique LSEs, where genes are only duplicated in one unique genome and there is no orthologous gene in any other five genomes. (2) Typical LSEs that are formed from a gene for which at least one ortholog is found in at least one other of the genomes studied. Table 1 summarizes the distributions of these two types of LSEs in *Plasmodium.*

The lineage-unique gene families are likely to have more impacts on the genome because they carry species-specific signatures and appear to be "novel" within the pan-genome. Our further analyses focused on this group of LSEs. Two rodent parasites, *P. chabaudi* and *P. berghei,* have the largest numbers of lineage-specific LSEs (111 and 176, respectively); this may simply reflect the fact that these two genomes were predicted to have much larger number of ORFs.

**Figure 1:**
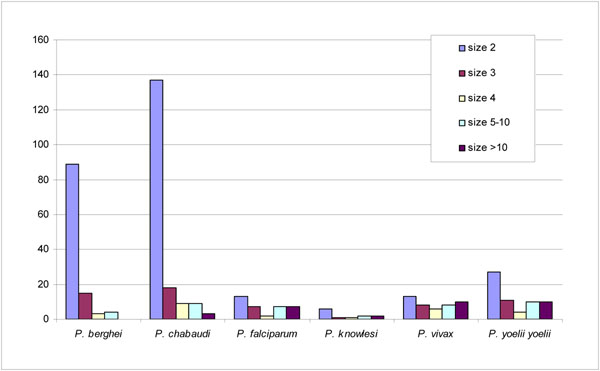
The distribution of the size of lineage-unique expanded multiple-gene families in *Plasmodium.*

The majority of these LSE families in *Plasmodium* contain only a small number (≤10 copies) of genes (Figure 1). The gene family size ranges from two to 165. In individual *Plasmodium* species, 28%-80% of the gene families are of size 2, and, collectively, gene families of 2-4 genes account for 60%-96% of the gene families. Large gene families are rare. The largest family is the rifin family in *P. falciparum* which has 165 paralogous members. Although the cellular function of rifins remains unknown, the antigenic variation in these proteins makes them vaccine candidates [36]. A recent phylogenetic and function shift analysis suggested that neo-functionalization and subfunctionilzation may have occurred during the rifin evolution [37]. Similarly, a complex evolutionary pattern is found in the second largest LSE in *P. falciparum,* erythrocyte membrane protein 1 (PfEMP1), another vaccine candidate which is proven responsible for antigenic variation and cytoadhesion of infected red blood cells [38,39].

It is extremely challenging to study the impact of LSEs in *Plasmodium* as most of these gene families are hypothetical proteins with unidentified functions - over 60% of the ORFs are annotated as hypothetical even in the best-studied *P.falciparum* genome [4]. For example, 85 out of 111 of the lineage-unique families in *P. berghei* were predicted to be hypothetical, and the rest of them were predicted to be "putative", "Pb-fam" or "BIR protein", with none being functionally characterized. In the genome of *P. chabaudi,* several clusters of genes were annotated as "cyclin-related, putative", however, no clear evidence supports this prediction.

Some of the lineage-unique LSEs may carry out functions or have distinct antigenic features, which may be related to characteristics of the host organism that distinguish it from the other *Plasmodium* species. For example, *vir* genes, *P. vivax* variant genes coding for variant antigens exposed on *P.* vivax-infected reticulocytes, can be classified into several subgroups based on their sequence and structural diversity [40]. Although antigenic variation is common in *Plasmodium* species as a mechanism for parasites to evade the host immune system, different parasites appear to evolve different surface antigens with tissue-specific activities. Vir, for instance, is implicated in spleen-specific cytoadherence in chronic infections [41]. Similarly, a group of seven paralogous genes are found in *P. knowlesi,* forming SICAvar-like antigen, the simian specific surface antigen.

There are also remote homologs of genes with potential functions. For example, two paralogous genes (PFI0115c and PFI0120c) are likely to be products of a recent gene duplication event as they are tandemly located next to each other on chromosome 9. They are annotated as "Serine/Threonine protein kinase, FIKK family", however, there is only weak statistical support (E-score = 0.00035) for the presence of a kinase domain in a PFAM domain search. It is unclear whether there is indeed a kinase activity in these putative proteins.

## Conclusions

Comparative genomic analysis of the six *Plasmodium* species with varying host specificity revealed 3,351 core genome components, whose functions range from fundamental biological processes to complex networks specific to a parasite-specific lifestyle. These core components represent the minimum requirement to maintain a successful life cycle that spans vertebrate hosts and mosquito vectors. They also include functionalities important to pathogenesis and adhesion to and invasion of host cells, indicating these six strains share a common mechanism for carrying out this phase of parasitic life cycle. Lineage specific expansions have given rise to abundant gene families in *Plasmodium.* Although functions of the majority of these families remain unknown, these LSEs could reveal components in parasite networks that, by their enhanced genetic variability, can be tied to pathogenesis, virulence, responses to environmental challenges, or interesting phenotypes.

## Methods

### Data

We collected the complete genomes of six *Plasmodium* species (Table 1) from PlasmoDB, the Plasmodium Genome resource center (http://www.plasmodb.org) [42]. The nucleotide, protein, and annotation data of Release 5.5 (September 29, 2008) were downloaded.

### Sequence similarity search and identification of orthologoues and paralogous families

To identify the presence of orthologous and paralogous genes, we pooled all the protein sequences from the six *Plasmodium* genomes and conducted an exhaustive all-against-all BLASTP search; genes were defined as orthologous or paralogous if (1) they had a FAST A E-score < e-10; (2) their similarity I was ≥30% if the length of the alignable region L ≥150 amino acid residues (or I = 0.01n + 4.8L(-0.32(1+exp(-L/1000))), if L <150 aa, where n = the number of sequences); (3) the length of the alignable region between the two sequences was >50% of the longer protein [43]; (4) Low complexity regions were filtered out.

A Markov cluster algorithm, OrthoMCL, was used to cluster genes into gene clusters [44]. The gene clusters contain the orthologous and paralogous genes from different genomes.

Multiple alignments of each cluster were obtained by the program ClustalX [45] and T-coffee [46], followed by manual inspection and editing. Phylogenetic trees were inferred by the neighbor-joining method, using MEGA4 [47]. The inferred phylogenetic relationships were used to detect the orthologous and paralogous genes in each cluster.

### Functional classification analysis

A hierarchical classification of cellular component, biological process, and molecular function was performed for each *Plasmodium* sequence by searching against the Gene Ontology database [48]. The classification of specific supergene families including transporters, kinases, and proteases was based on the standard nomenclature defined in the Transporter Classification (TC) system [49], the Kinase Classification System [50], and the Merops Peptidase Database [51].

## List of abbreviations used

DOXP: 1-deoxy-D-xylulose 5-phosphate; GO: Gene Ontology; GPCR: G-protein coupled receptor; LSE: lineage-specific expansion; MSP: Merozoite Surface Protein; ORF: open reading frame; PfEMP: *P. falciparum* erythrocyte membrane protein; RBC: red blood cell; VBEM: variational Bayesian expectation maximization; TC: Transporter Classification

## Competing Interests

The authors declare that they have no competing interests.

## Authors' contributions

YW, JG, and HC conceived and designed the study. They all performed data analysis. YW and HC drafted the manuscript. All authors read and approved the final manuscript.

## Supplementary Material

Additional file 1 A core genome of six Plasmodium genomes comprised of 3,351 orthologous groups is listed. Brief descriptions of predicted gene functions and GO functional classification are also included. Click here for file

Additional file 2 The lineage-unique and typical LSEs are presented in the second and third spreadsheets, respectively. Click here for file
